# Imaging and seismic modelling inside volcanoes using machine learning

**DOI:** 10.1038/s41598-023-27738-6

**Published:** 2023-01-12

**Authors:** Gareth Shane O’Brien, Christopher J. Bean, Hugo Meiland, Philipp Witte

**Affiliations:** 1 Microsoft Ireland, Dublin, Ireland; 2 Geophysics Section, Dublin Institute for Advanced Studies, Dublin, Ireland; 3 Microsoft Netherlands, Amsterdam, The Netherlands; 4 Microsoft United States, Redmond, USA

**Keywords:** Solid Earth sciences, Seismology, Volcanology

## Abstract

Despite advances in seismology and computing, the ability to image subsurface volcanic environments is poor, limiting our understanding of the overall workings of volcanic systems. This is related to substantive structural heterogeneities which strongly scatters seismic waves obscuring the ballistic arrivals normally used in seismology for wave velocity determination. Here we address this constraint by, using a deep learning approach, a Fourier neural operator (FNO), to model and invert seismic signals in volcanic settings. The FNO is trained using 40,000+ simulations of elastic wave propagation through complex volcano models, and includes the full scattered wavefield. Once trained, the forward network is used to predict elastic wave propagation and is shown to accurately reproduce the seismic wavefield. The FNO is also trained to predict heterogeneous velocity models given a limited set of input seismograms. It is shown to capture details of the complex velocity structure that lie far outside the ability of current methods available in volcano imagery.

## Introduction

Volcanoes are some of the most enigmatic objects in Earth science. They emit a diverse variety of seismic signals with high rates. These volcanic seismic signals have long been items of study as they carry information about the physical processes inside volcanoes and can exhibit different characteristics to seismograms generated from tectonic earthquakes^1–3^. The different signals observed include long-period events, very long-period signals and tremor and have been linked with a range of different physical source mechanisms^4–9^. The event locations and source mechanism are key constraints on the possible physical processes that can explain these signals and hence are critical pieces of information in determining the hazard potential of volcanoes. A vast number of studies have been undertaken to determine the location and source mechanisms of volcano seismic signals which are then related to the underlying source processes^10–14^. However, the outstanding barrier faced in determining robust answers from these studies is the requirement for a detailed subsurface velocity model, so that the distorting effect of highly heterogeneous volcano structure can be removed from the field recorded seismograms. Numerical simulations using representative volcano velocity models have demonstrated a very strong influence of wave propagation path effects on the inversion results for both the source location and source mechanism^15^. Also, short duration source wavelets can exhibit large train long-duration waveforms at distal seismic stations as a result of wave propagation through layered strata rather than source related components^16^. Shallow magma has even been drilled by accident in a geothermal field, due to the failures of geophysical imagery in these complex environments^17^. Improved seismic imagery is required to determine the geometry of sill intrusions and the boundaries of possible magma chambers. High resolution seismic velocity imaging is arguable one of the most challenging problem to solve in volcano seismology. Velocity models derived from passive earthquake tomography are low frequency and also smooth out structures along ray paths. Hence they do not contain the desired geological structure that would account for the strong wave scattering path effects observed in volcanic seismic signals^18^, as scattering relates to the spatial gradient of the velocity field. There are very few successfully resolved high resolution seismic models available in volcanic environments unlike in exploration reflection seismic experiments in sedimentary basins Although there has been some success at imaging exceptionally reflective individual discrete structures^18,19^. Generally on volcanoes, strong incoherent wave scattering masks coherent reflections with resultant imaging problems limiting access to high resolution structural and velocity information even though the information about the path effects, and hence velocity structure is inherently encoded into the recorded seismograms. The central problem is that the dominant recorded wavefield is not sufficiently correlatable to allow the reconstruction of the underlying structure nor its velocity field. Here, we present a new paradigm in attacking the velocity inversion problem in volcanic environments. We take a deep learning approach using a form of neural networks both to model and to invert complex volcano seismic signals. An alternative to the traditional geophysical inversion techniques is to consider the data inversion task as an optimisation problem and deploy recent advances in machine learning. The central idea is to use the entire scattered wavefield to address the imaging problem—not merely events that are coherently reflected off spatially correlatable geological structures. The accuracy of these machine learning techniques, specifically deep neural networks, has dramatically increased across nearly every field of research^20^. Recent advances in machine learning can be attributed to the increase in computer power and the adoption of deep-learning methods^21^. In geophysics, machine learning has been long established^22,23^, however, a suite of recent applications has appeared in the literature. These include seismic processing, automatic fault detection, noise suppression, micro-seismic detection, as well as diffraction identification^24–29^. Here we deploy a special type of neural network called a Fourier Neural Operator (FNO) which has been proposed to predict the results from a partial differential equation (operator) given an input model and initial conditions^30^. In our case the network predicts the Green’s functions for a specific equation and hence the reverse network is capable of generating the inverse Green’s functions which is the kernel for velocity inversion. This study assesses the problem from a theoretical viewpoint focusing on the question: can neural networks accurately model and invert seismic wavefields in a complex volcanic environment?

## Methods

### Fourier neural networks

A partial differential equation (PDE) can be viewed as a differential operator ***L*** mapping a function ***g*** to the function ***f***, i.e. ***Lg*** = ***f***. This is the forward problem where the operator ***L*** in this case is the elastic wave equation. The inverse case is where the inverse operator ***L***^−**1**^ maps the function ***f*** to the function ***g***. We can automatically link the operator equation with the equation ***Gm*** = ***d*** where the elastodynamic Green’s functions ***G*** for a given Earth model ***m*** produce the seismic data ***d*** and the inverse relation ***G***^−**1**^***.d*** = **m** generates an Earth model **m** given the data and the inverse Green’s functions, ***G***^−**1**^. In our specific case, the application of ***L*** determines ***G*** for a specific model **m**. Neural networks are a mapping from an input vector space to an output vector space or an input image vector to an output image vector which is an equivalent representation of the differential operator above and, in theory, can learn to predict the operator or inverse operator given appropriate input training data. The FNO network was the first network to model turbulent flows and was used to predict results from the Navier–Stokes equation, Burger’s equation and Darcy’s equations^28^. Recently, it has been used to predict two phase flow in the subsurface given a subsurface model and initial conditions^31^. The FNO has some distinct advantages over other networks such as convolution neural networks (CNN) that make it readily adaptable to learning differential operators. The method is resolution and mesh invariant hence it can take a low resolution input training dataset and predict higher resolution outputs on arbitrary meshes. The convolution in the CNN is replaced by a multiplication in the Fourier domain and this has some advantages. The first is the compute complexity of a convolution versus the fast Fourier transform, making the FNO more computationally efficient. PDEs are continuous and the global nature of the Fourier space is more suited to capturing this continuity versus the local convolution kernels in a CNN. Additionally, higher frequencies can be truncated without significantly impacting the solution, which is in essence a compression routine, increasing the compute efficiency. In practical terms for this work, large volumes of synthetic seismic data are generated using a partial differential solver given an input model. This can be considered as the ‘observed’ data. The FNO is trained using this dataset for both the forward and inverse problem. When subsequently applying the trained FNO for the forward problem, the network takes the models as input and predicts the expected elastic wavefields and synthetic seismograms. When applying the FNO for the inversion network, the input is the ‘observed’ seismograms from a previously unseen simulation in a specific earth model of interest (equivalent to observed data), and the prediction is that specific velocity model. The FNO prediction output is orders of magnitude faster than running a PDE solver, but the generation of the training data and the FNO training itself does incur a significant upfront computational cost, dependent on the problem.

### Synthetic data generation (‘the observations’)

By definition there is no physical dataset which can be used to train the FNO system to invert seismograms for a known subsurface velocity model. Supervised FNO training requires solutions to the wave-equation as inputs. Therefore we need to generate training datasets using known input velocity models, source locations and source mechanisms and use a forward wave equation solver to output training sets comprising seismic wavefields and synthetic seismograms. Given the heterogeneous nature of volcanic materials we need to choose a solver that can propagate seismic elastic waves in materials with broadband heterogeneity with strong velocity gradients. Here we chose a 2D elastic lattice method^32^ as a full wavefield simulator. We generate two different training datasets and one independent test dataset. It is stressed that the test data are never used in the training of the networks. The generation of synthetic datasets requires several choices to be made which constrain the solution as without such constraints, the parameter space is too large for practical implementation. The assumptions made in the generation of the datasets are detailed below.

#### 2D models

Although there is a recent example in acoustic wave applications^33^, we are not aware of previous work which attempts to use the FNO to learn the elastic wave equation Green’s function and its inverse, for a complex model. In order to explore the feasibility of this approach in complex media, we focused on 2D examples which reduce the computational demand and allows us to constrain the approximate cost and challenges associated with producing reasonable results. Future moves to 3D will increase the computational cost in generating the synthetic datasets and in training the FNO along with the need to use a larger neural network.

#### Source

The source locations were chosen at random anywhere inside the 2D model but the depth was constrained to be less than 6 km. The source mechanism was the moment tensor (m_xx_, m_zz_, m_xz_) where component values were randomly chosen between 0 and 1 for the diagonal components and 0 and 0.5 for m_xz_. The source wavelet for all simulations was a Ricker 4 Hz wavelet. This was chosen as it overlaps with the long period volcanic signals typically observed in such settings. The higher the frequency, the higher the spatial resolution required and hence the computational cost grows for both the simulations and the FNO training.

#### Training velocity models

**Figure 1:**
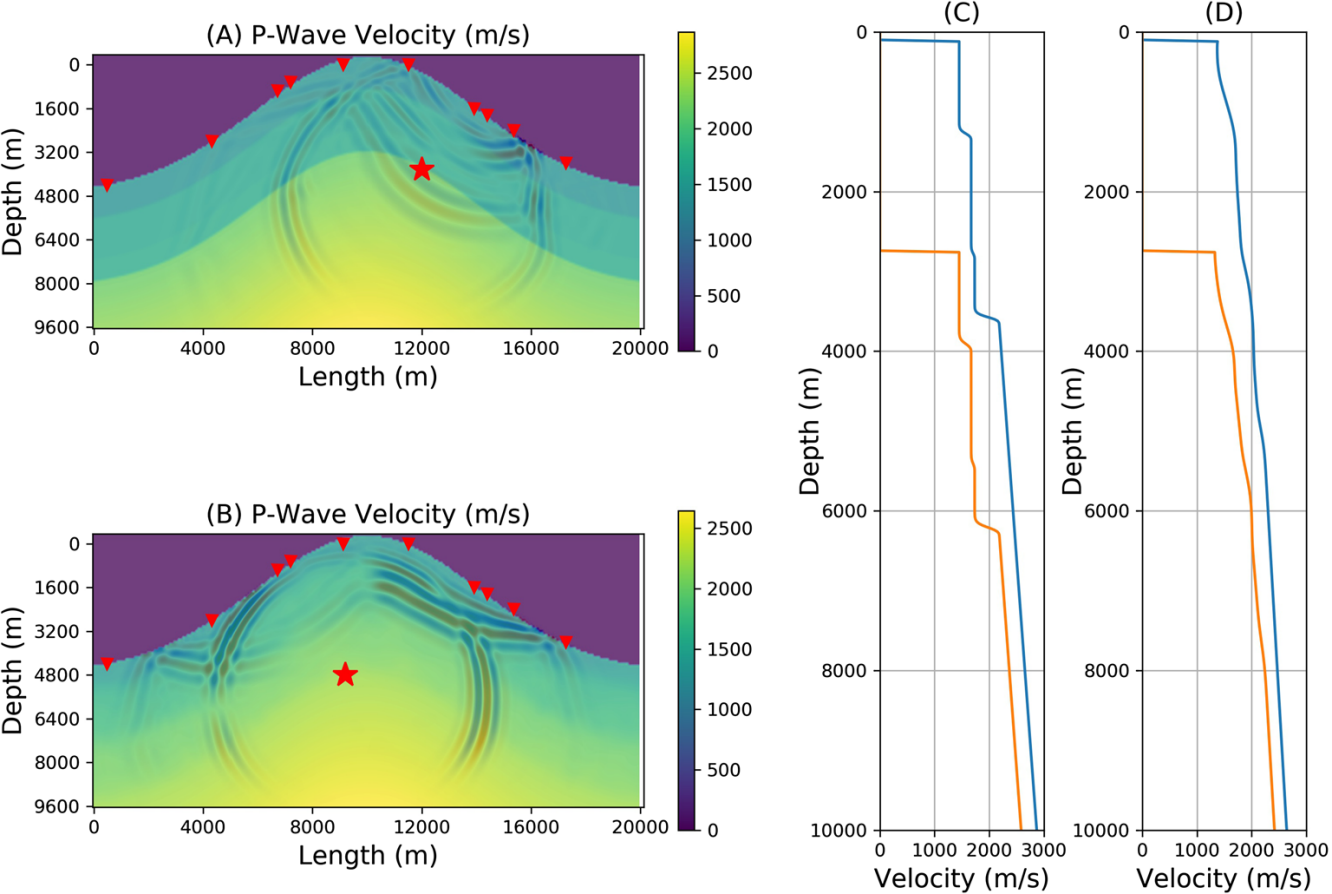
(**A**) A single p-wave velocity model drawn from population L which consists of 20,000 velocity models. The numerically simulated seismic wavefield using an elastic lattice wave propagation method is shown overlain on the models with the source location (shown by the *). (**B**) A single p-wave velocity model drawn from the population T which consists of 20,000 velocity models. The numerically simulated seismic wavefield is shown overlain on the models. (**C**) The velocity profile through the model shown in (**A**) from two different locations in this model. (**D**) The velocity profile through the model shown in (**B**) from two different locations in this model. The triangles in (**A**) and (**B**) show the location of the synthetic seismic stations at which time varying synthetic seismic data are ‘captured’ during the simulation run.

As Neural Networks are poor at extrapolation, for any real world application the choice of training set is a key consideration. The dominant characteristics of the expected physical structure must be included, in a general framework, in the training set. Here we utilise two different velocity model populations, with different statistical distributions to generate synthetics for FNO network training. Due to their formation history the mechanical complexity of volcano edifices means that a deterministic calculation of their fine scale structure is out of reach, and a statistical approach is more appropriate. Here the topography was fixed for all model populations as a Gaussian hill, as topography is one of the only parameters that can be accurately measured in real environments. The first velocity model population, denoted as population L, has a p-wave velocity gradient randomly chosen within the bounds of a minimum velocity of 1500 m/s and a maximum of 3500 m/s. Four layers, tracking the topography, were superimposed on top of this gradient, where the thickness of the layers were randomly chosen and velocities randomly perturbed from the gradient value. The s-wave velocity was set to two thirds the p-wave value. In the second model population, the same velocity gradient was used but the velocity field was randomly perturbed with a normal distribution and smoothed to yield a tomography-like velocity. This population is denoted as population T. Both populations L and T are each composed of 20,000 individual velocity models used to generate the synthetic seismograms for training the FNO networks and a further 500 velocity models for testing the networks. The test models in the populations are not used in training. Two sample models, drawn from the populations L and T, are shown in Fig. 1. By restricting the study to these two populations, we expect the network to be only able to learn how to generate wavefields from models drawn from these distributions or return velocity models within these distributions. In a real world scenario, the solution to the problem of constraining an input velocity distribution could be to take known geological constraints on the possible structures inside a particular volcano and build a large distribution of representative geological velocity models with the appropriate topography. This is the approach that will be taken for a full 3D inversion of real data in future studies.

#### Validation velocity models

Another population of models was created to validate the approach but was never used to train any network. This population, denoted population V, is similar to population T but without the smoothening and with a higher magnitude velocity perturbation.

#### Simulation parameters

**Figure 2:**
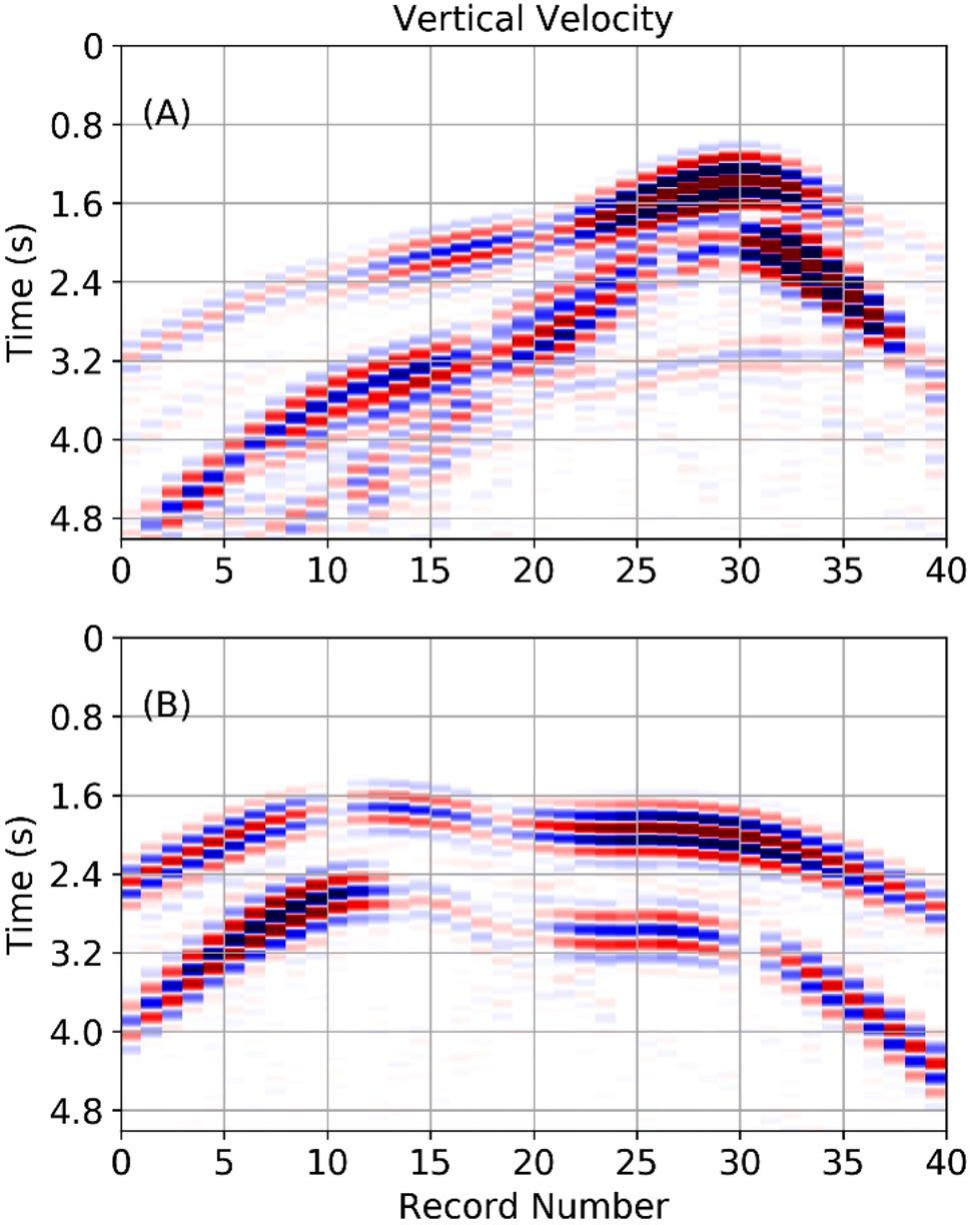
40 synthetic seismic sensors are distributed across the surface of all models. The seismogram amplitudes are normalised. This example shows synthetic vertical seismic traces for the sample from population L in panel (**A**) and population T in panel (**B**). See also Fig. 1. A random selection of 10 sensors is used in FNO training.

The model size was 20 km in length and 10 km deep with a spatial sampling of 10 m. The output temporal resolution of the synthetic seismograms was 1 ms. Output seismograms are 5 s long. Absorbing boundaries were imposed on the side and bottom to avoid unrealistic reflections. Each individual simulation for one model outputs the entire wavefield, both the x- and z-components of the displacement every 0.1 s along with synthetic seismograms. Figure 1, panels (A) and (B) shows one snapshot of the wavefield overlain on the velocity model. The location of the stations are shown in Fig. 1 as triangles on the surface of the model volcano. These locations were randomly selected sensors from the full synthetic sensor network of 40 sensors disturbed evenly across the full model. We randomly selected 10 sensors from 40 as having a dense full azimuthal coverage is rarely the case in physical world volcano settings. Adding more sensors, adds more information and increases the training computational requirements but the additional information can help increase the accuracy of the network. Figure 2 shows the synthetic seismograms from all 40 sensors for both models simulated using the elastic wave equation solver.

### Training

**Figure 3:**
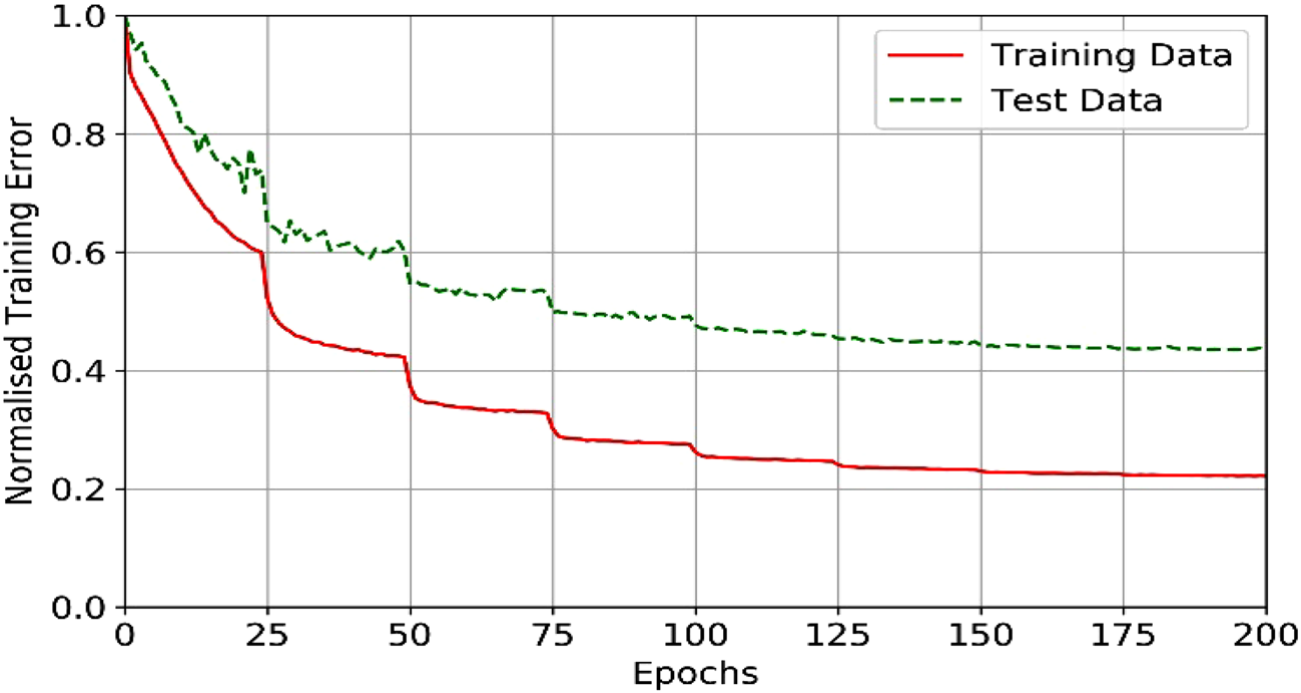
Training error for one of the network learning runs. Here the training data were generated using 20,000 simulations and 500 simulations used for the test data.

Each sample from model populations L and T with a random source location and a random source mechanism is used as inputs to the 2D elastic wave simulator and the resultant synthetic seismograms and wavefields are outputted and saved. Therefore, the training datasets consist of the velocity models from the populations L and T, the source locations and the synthetic wavefields and seismograms. The training datasets comprise a total of 40,000 numerical seismic wavefield forward propagator simulations (20 k for each model population) and the model populations L and T. These training datasets are used to teach the several different FNO networks presented in the results. We will denote the training datasets using the same terminology as the L and T model populations. In order to train the FNO to act as a fast forward wavefield propagator (i.e. to output wavefields and seismograms), it was trained using the source locations, the source mechanisms and the velocity models as inputs. To train the FNO to act as an inverse modelling tool (i.e. to output a velocity model) the training datasets have the inputs and outputs switched and the networked mirrored. The results for four networks are presented in this work. The first two are the forward modelling networks for both training datasets L and T. The second pair are for the inversion networks for both the training datasets L and T. The networks are four layers deep and built using PyTorch^34^ (version 1.9.0). For both inverse and forward networks, as discussed above, the training data consists of 20,000 samples with a batch size of 20 run over 200 epochs. A variable learning rate was imposed with a 50% reduction after 25 epochs. The input data were normalised with a Gaussian normalisation function and the optimiser was the AdamW algorithm though the choice did not have a significant impact on the results. These parameters were found by trial and error on initial tests balancing the amount of data required to provide reasonable results against the computational time. Figure 3 shows the normalised training error for training the layered inversion network. This is typical of all the training runs. The test data which consists of 500 samples, was not used in the training.

The main parameters controlling the training were the size of the training samples, the complexity in the velocity models and the number of epochs. Initially, tests on a homogenous model distribution (not shown here) required at least 5000 training samples before a reasonable training error was attained after 100 epochs. For the more complex velocity model populations T and L, the number of samples needed to be increased towards 20,000. A test with 40,000 sample simulations for population L did not significantly improve the results over the 20,000 samples and started to show the typical signs of overfitting in Neural Networks. The accuracy of the results on the test data is discussed in the “Results” section.

## Results

### Forward modelling results

**Figure 4:**
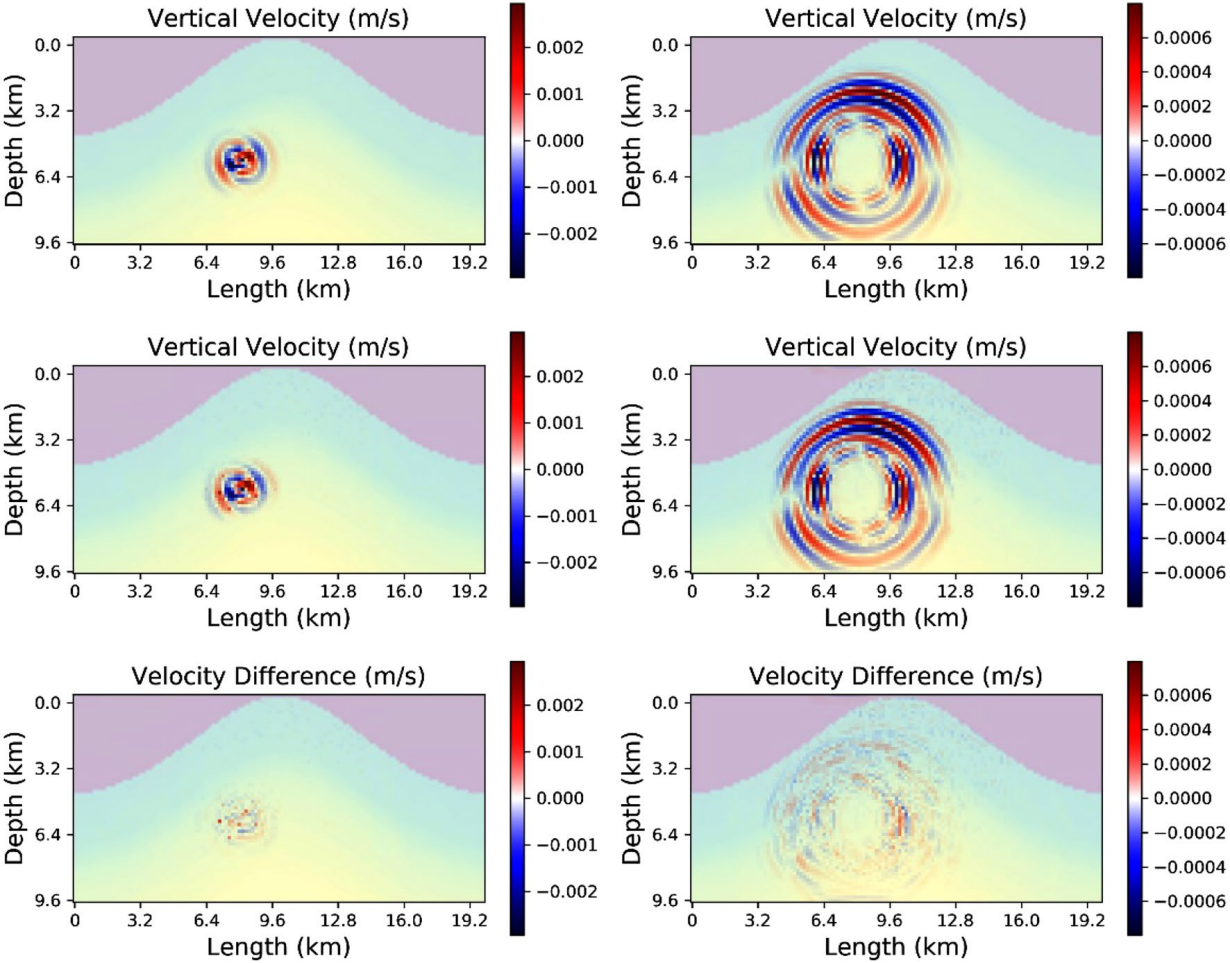
The top panel shows the results from a numerical simulation directly solving the elastic wave equation with a sample from the population L which was not used during the training. The left-hand side is for a snapshot after 1 s and the right-hand panel is after 3 s. The middle panel shows the prediction from the FNO network. The bottom panels show the difference.

Before we consider the application of the FNO to the classical geophysical inversion problem of resolving an accurate velocity model given seismic data, we will demonstrate the ability of the FNO to simulate wavefields and seismograms, i.e. the forward problem. The inputs are the velocity models, the source location and the source mechanism. The outputs are the seismic wavefields and/or the seismograms. The seismograms are a spatial subset of the total wavefields, captured at the model top surface. The output wavefield for two different time periods produced by using the elastic equation solver is shown in the top panels in Fig. 4. The model used in this case is a single example from test data from population L and when input into the appropriate FNO network predicts the output wavefield, middle panels. The comparison between the two show that the FNO system does an excellent job at replicating the main features of the wavefield. This is confirmed by the bottom panel plots where the difference between the snapshots is presented. The accuracy is not at the level one would expect when comparing two different explicit solvers to the elastic wave equation, but the dominant high amplitude phases in the wavefield are captured with the correct amplitudes. The FNO prediction equally resolves the main p-wave and s-wave phases as seen in the 3 s snapshots. The results for network trained using the training dataset T are similar. As the full wavefield can never be physically recorded in practice, a more useful approach would be to focus on the seismograms from known locations. This has the added benefit of decreasing the computational cost as the input data volumes are smaller, as are the output layers in the network.

**Figure 5:**
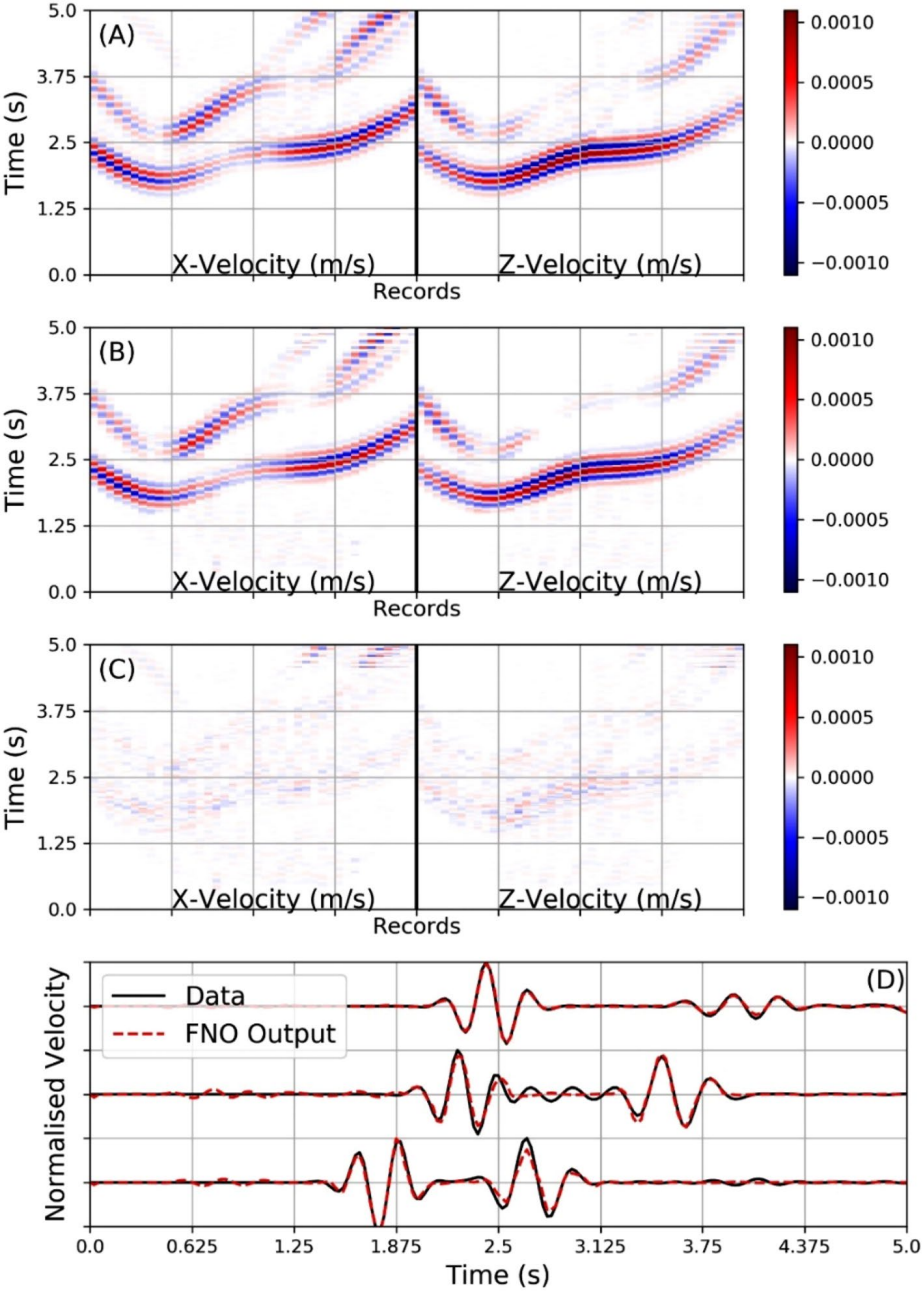
The top panel (**A**) shows the results from a numerical simulation directly solving the elastic wave equation with a previously unseen sample model from population L. Panel (**B**) shows the seismogram prediction results from the FNO network (previously trained with surface seismic data only) when presented with the same layered model as in panel (**A**). Panel (**C**) show the difference between the simulation traces and the predicted FNO network outputs. Again the overall match is very good with all main arrivals captured. There are some discrepancies associated with small amplitude scattering. Some edge artefacts from the training set-up can also be observed. The bottom panel (**D**) shows three simulated traces compared against the FNO predictions, demonstrating a very good match in the main.

Figure 5 shows the results, where instead of using the entire wavefields to train the network, we only use the surface seismogram data, in this case all 40 sensors. The top panel in Fig. 5 shows the simulated outputs from the direct solver and the middle panel shows the prediction from the FNO trained with population L; the bottom panel shows the difference between the top two panels. Again the FNO demonstrates an excellent ability to capture the main features of the wavefield when employed as a forward propagator. This is despite the limited coverage of merely surface seismic records as input data for training. This bodes well for real world applications, where usually only surface seismograms are available. The difference between the two do show some edge artefacts in the neural network output along with small scale misfits in the small amplitude scattering events. Several reversible normalisation methods, including time and log normalisation, were tested to determine if these smaller amplitude effects could be better predicted but with limited additional success. The amount of training samples does play an important role here but as discussed in the training section, the training and test error plateaus out after 20,000 training samples. Obviously the more complex the velocity model the larger the scattering which will diminish the ability of the FNO to predict all of the details in the seismograms. However, for the low frequency components of volcanic signals, the wavelengths can be on the order of 100’s of metres hence these smallest scale heterogeneities will ‘smooth out’. This is important to consider when interpreting the inversion results in the next section as the main amplitude phases which are accurately predicted can contain enough information to reproduce the velocity model.

### Inversion results (velocity model recovery from seismic data)

**Figure 6:**
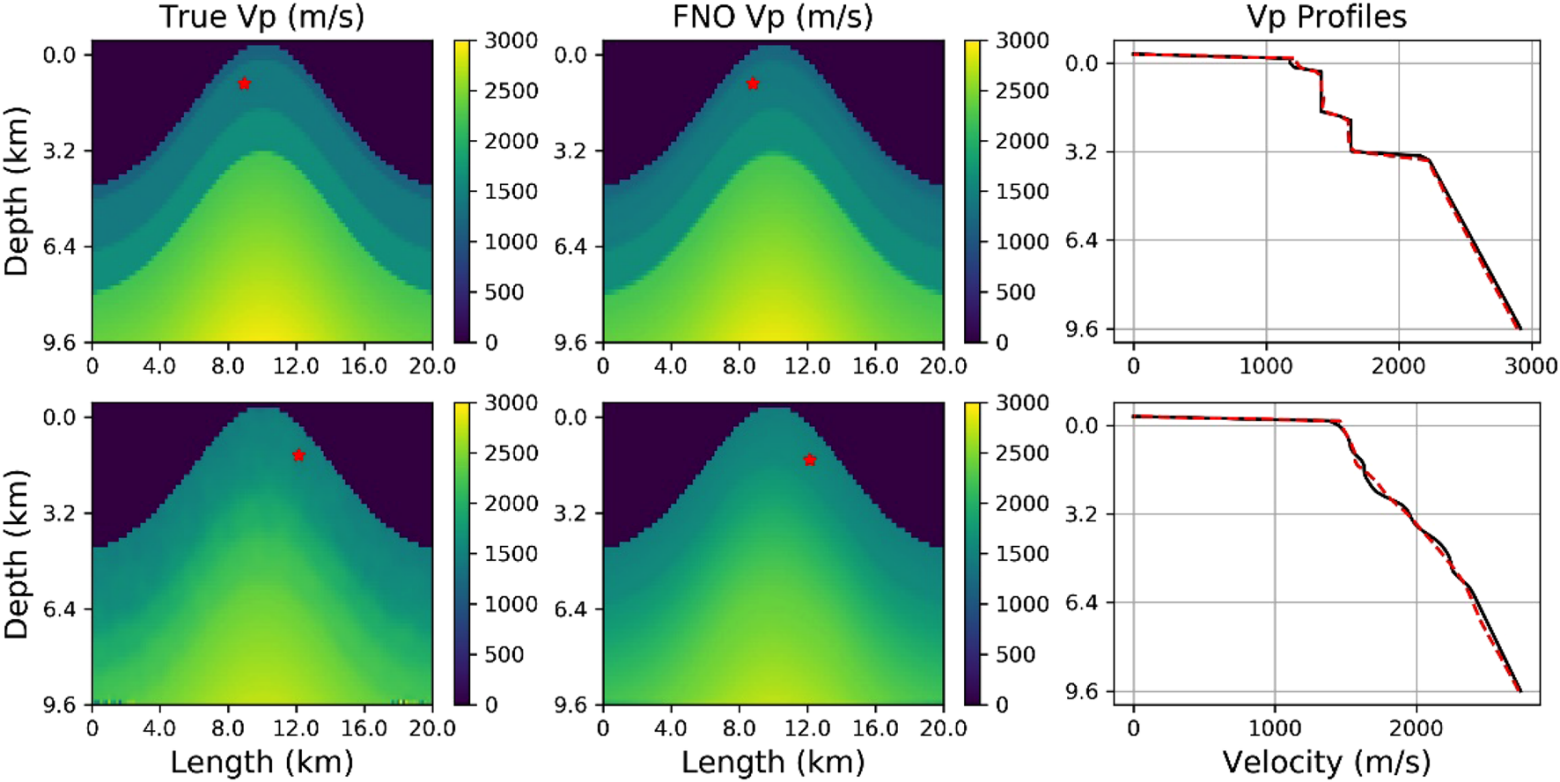
Left panels, the true unseen models velocity models (top panel from population L, bottom from population T) used to generate the input seismograms for the FNO network. Middle panels, the velocity model predictions (outputs from the FNO). Right panel, profiles through the true and predicted models. The seismic source location is highlighted by the asterisks. In the FNO output, the source location is also predicted by the network. Both velocity models here are each recovered from input data associated with only one randomly located shot into 10 randomly located surface receivers, each.

For the velocity inversion problem, the training was performed in exactly the same way as described for the forward problem and in the training section, except for two changes. The first difference was to reverse the inputs and outputs and adjust the layers to match the input sizes, i.e. mirror the network. The second difference was that when training the inversion FNO network the inputs were 10 randomly located seismograms, from the 40 described above, for both the x-component and z-component of seismic displacement. In all cases, white noise amplitudes of 15% were added to the input seismic traces. Inputs also included the known velocity model for each seismogram set and the source location. Following training, during the application of the inversion FNO the input is the set of 10 two-component seismograms (i.e. two individual shot gathers) from a previously unseen model. The FNO output is the predicted velocity model and the source location. In Fig. 6, examples of an input model from populations L and T are shown to illustrate the results. These are representative of all results, and all results shown use samples from the different populations that were not used in training the networks. The left-hand panels show the true models with the seismic source location highlighted by the asterisks. The output results from the FNO networks is shown in the middle panels, i.e. the network velocity model and source location prediction. The right-hand panels show a profile through the models comparing the true model with the predicted FNO result. It is clear from Fig. 6 that the FNO method is capable of accurately predicting a velocity model where the input is unseen surface seismograms generated in the unseen model (taken from populations L or T).

**Figure 7:**
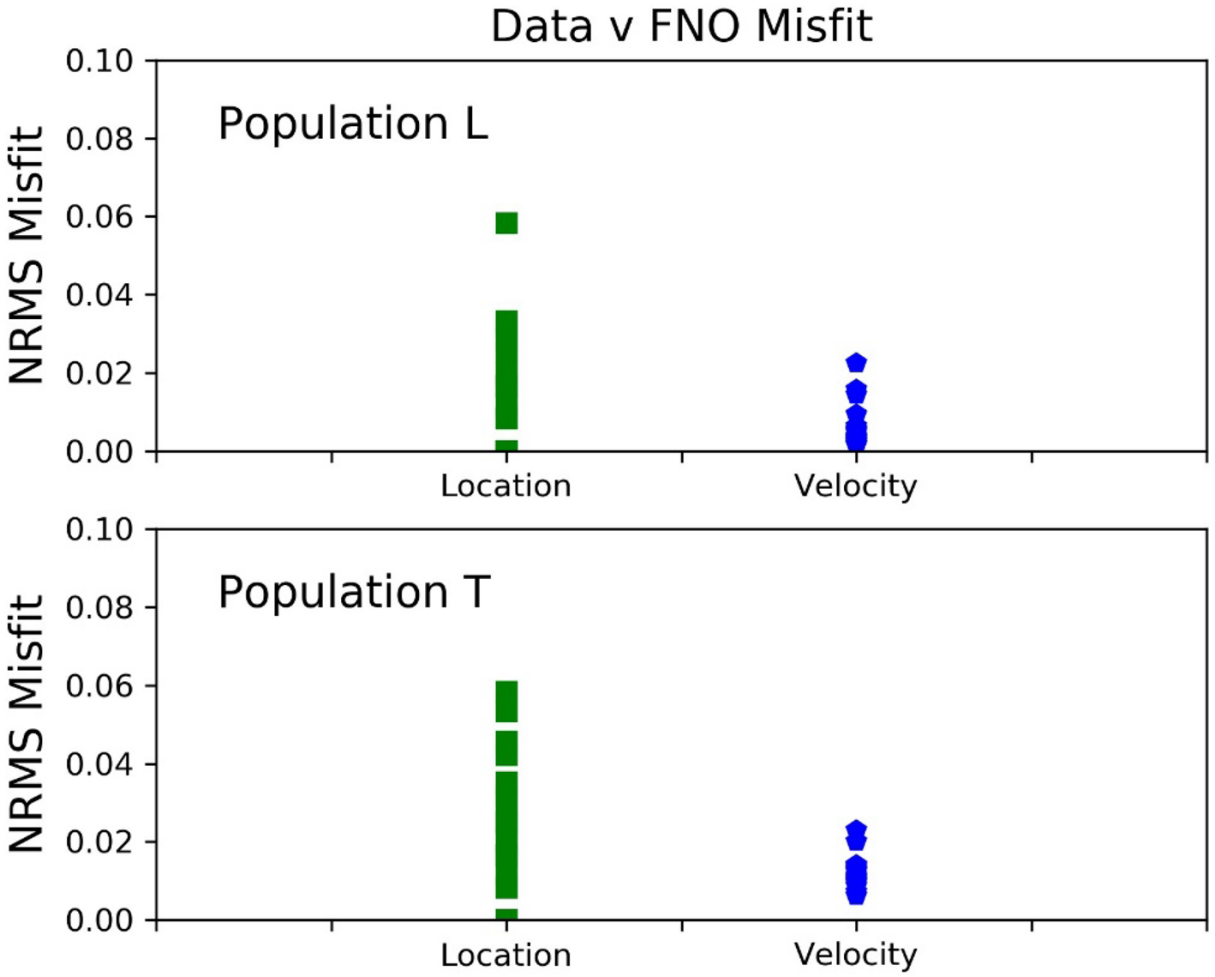
The normalised RMS misfit is shown for 20 different inversions using unseen samples from the population L in the upper panel and population T in the lower panel. 15% white noise was added to the input seismic traces during training. The velocity models are well resolved by the FNO inversions as are the source locations. All 20 inversions are for different previously unseen models with the same statistical properties as the training velocity model sets.

In order to quantify the results, the normalised RMS misfit between the true model inputs and the FNO generated outputs are shown for 20 different inversions using unseen samples from both populations L and T. The source location misfit is the difference between the (x, z) locations for true value and output value. The velocity normalised RMS is the sum over all velocity differences between the true and predicted model for every grid point in the model. The RMS values are shown in Fig. 7 for both populations L and T. The error in the velocity model is relatively small, and as illustrated in the velocity field comparisons and profiles in Fig. 6, the velocity model prediction is very good and is also relatively robust to the addition of noise in the training dataset up to the maximum value of 15%. The source location is also very good but more sensitive to the addition of noise. Several different normalisation strategies were explored to understand and improve the source error. The results pointing to higher accuracy for the velocity model and increased error for the location appear robust. In practise, this is less of a concern as traditional methods to determine the source location are well known and accurate even in the presence of a long wavelength velocity structure. As discussed in the previous section, the FNO network was used to predict the output from the elastic wave equation, in essence learning how to reproduce the Green’s function for such systems. The inverse network can therefore be considered to have encoded the inverse Green’s function for the wave equation and therefore allows for the prediction of an accurate velocity model following the analogous equation ***G***^−**1**^***.d*** = **m**.

**Figure 8:**
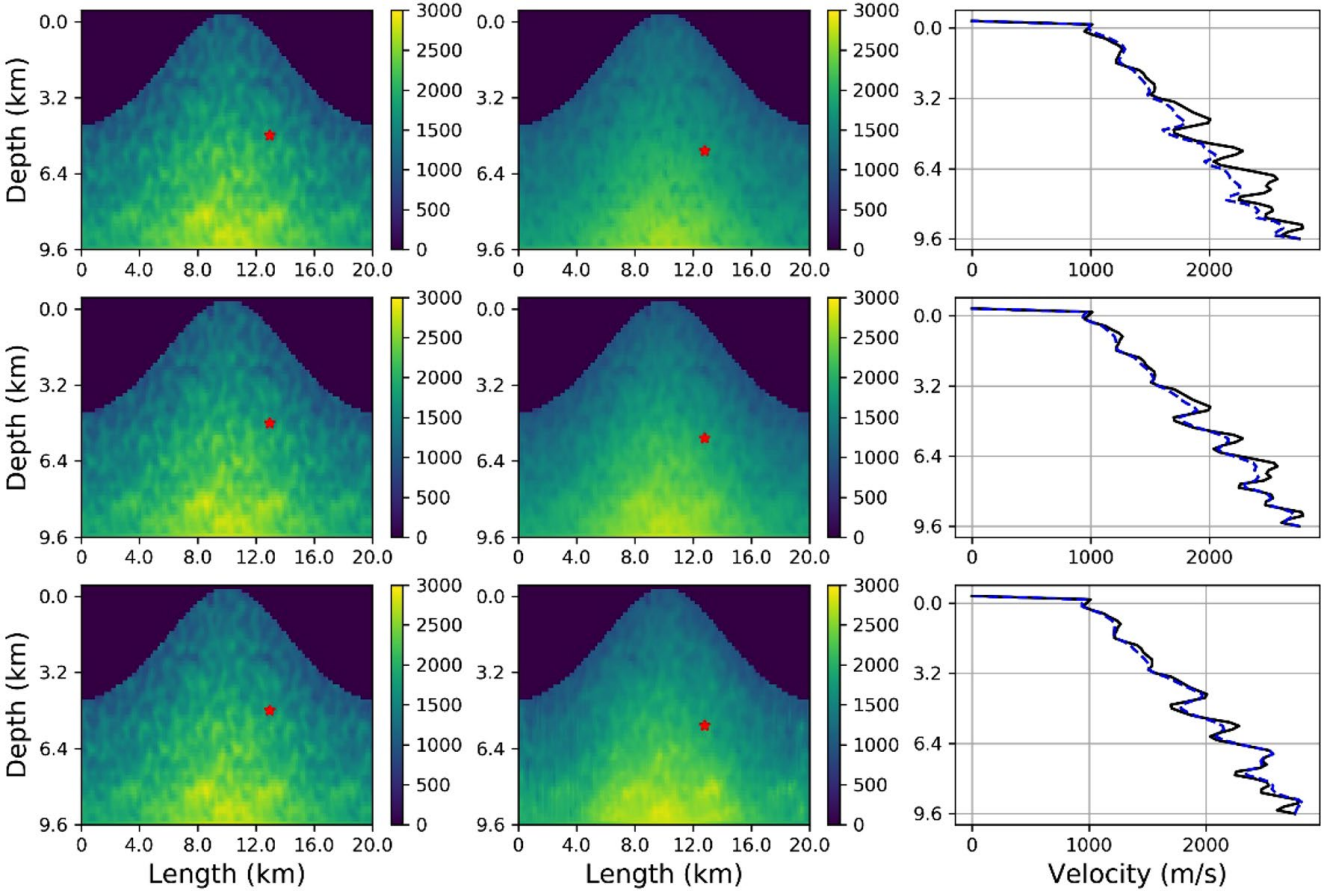
Top panels show from left to right, the true velocity model taken from population V, the FNO inversion result using synthetic seismograms from one shot gather (one source and associated seismograms), and a comparison of the velocity profiles. The FNO network was trained using seismic data simulated in velocity models drawn from population T. The middle panels replicates the top panels except the FNO result is the sum of 20 different inversions for different sources in different locations, using the same model, i.e. stacking velocity models FNO predicted from 20 shot gathers with randomly located sources. The bottom panels show results after stacking 100 predicted velocity models. The right hand panels clearly show the convergence to an accurate velocity model after stacking multiple velocity model predictions derived from different shot gather FNO inputs, with the true model shown by the solid line and the prediction by the dashed line.

The examples above show the results where input seismic data presented to the FNO were calculated in previously unseen velocity models with the same statistical properties as the velocity models used to generate the FNOs training seismic data. The input was a single shot gather with a random source location, in each case. We now show a final example where we use seismograms generated using a simulation in velocity models from population V as the input data to a FNO network that was trained using seismic data from velocity models drawn from population T. The prediction is from a single set of seismograms from a single source and one velocity model sample from population V, i.e. a shot gather. The top panel in Fig. 8 shows that this does not accurately reproduce the known velocity model. This is not a surprise as Neural Networks can struggle to generalisation across different training populations. However Fig. 8 does show that some features are recovered so we opt to stack the predictions from multiple shot gathers (random source locations, and their seismograms), in the same velocity model. This is shown in the middle and bottom panels in Fig. 8 where we stack velocity model predictions from 20 and 100 shot gathers respectively. The convergence to the true model is clearly demonstrated showing that even with a velocity model population that differs in statistical detail from the training populations, an accurate velocity model can be recovered through predicted velocity model stacking. Despite this success, it should be stressed that the appropriate choice of statistical parameters of the model populations using in FNO training will play a dominate role in the ability of networks to converge to a solution. It still is an open question as to how to determine the statistical properties of the training dataset to maximise the ability of the network to converge to a general solution or even if this will be possible given the extreme heterogeneity in velocity structure in volcanic environments in real world scenarios. We propose that a priori information such as topography, the population of rock physical properties, borehole petrophysical log information etc. could effectively be used to geologically constrain the ‘bandwidth’ of training velocity model populations, for a given volcanic environment. Overall, the results for both the inversion and forward problem highlight the applicability of the FNO network to solve a highly non-linear inversion problem in elastically heterogeneous environments, and to replicate the seismic wavefield for rapid forward calculations.

## Discussion

The FNO can take unseen synthetic data and discover the seismic velocity model and seismic source that would generate this dataset, if using a traditional PDE seismic wavefield forward solver. It can also be used to simulate synthetic data given an input model that is accurate when compared with a traditional PDE solver, except for the low amplitude scattering. The objective is not to replace traditional solvers as they are required for the generation of the training data. As seismic imaging on volcanoes is still a huge challenge we suggest that the inverse problem is of more interest, as this may allow access to the true velocity structure when the input to the Neural Network is real data. A challenge with using real data as inputs into these networks may centre on sufficient environmental noise samples in network training, but fortunately this can be captured statistically^35^. A more challenging question is how to build a population of numerical training models that sufficiently represent the physical world? The requirement is that such a numerically trained FNO will return an accurate representation of real world velocity structure, when presented with appropriate real field seismic data. Here, in this work, we demonstrate that if that training model population building challenge can be overcome, then the FNO has a high probably of accurately inverting for real world velocity structure. The details of how this might be achieved will be the focus of future work, but it will likely require the building of hybrid statistical-deterministic models, based on high spatial resolution mapping. The computational scaling to 3D remains a significant challenge, not in the prediction, but in the generation of a significant number of input datasets. Whilst this will require large resources, the resolution invariance of the FNO means lower resolution training data can be used and still provide higher resolution predictions. This theoretical study has demonstrated the viability of deploying machine learning tools as a fresh approach to the arduous task of deriving high fidelity velocity models in volcanic environments. If such models can be obtained they will, for example, increase our understanding of how seismic sources relate to the specifics of how magma is emplaced. It would also allow local volcano observatory based real time tracking of magma migration through repeated imagery with ease, as the forward FNO is fast and computationally efficient. Such a routine capability could radically impact risk mitigation on volcanoes.

## Data Availability

All the data used were generated by the computer codes discussed in the text. The datasets generated and/or analysed during the current study are not publicly available due to the size of the datasets but can be recomputed following the method outlined in the manuscript.

## References

[1] McNutt SR (2005). Volcanic seismology. Annu. Rev. Earth Planet. Sci..

[2] Wassermann, J. in *IASPEI New Manual of Seismological Observatory Practice (NMSOP)* Vol. 1 (ed. Bormann, P.) (GeoForschungsZentrum Potsdam, 2002).

[3] Chouet, B. A. in *Volcanic Seismology* (eds Gasparini, P., Scarpa, R. & Aki, K.) 133–156 (Springer, 1992).

[4] Chouet B, Julian BR (1985). Dynamic of an expanding fluid-filled crack. J. Geophys. Res..

[5] Neuberg J, Luckett R, Baptie V, Olsen K (2000). Models of tremor and low-frequency earthquake swarms on Montserrat. J. Volcanol. Geotherm. Res..

[6] Jousset P, Neuberg J, Sturton S (2003). Modelling the time-dependent frequency content of low-frequency volcanic earthquakes. J. Volcanol. Geotherm. Res..

[7] Chouet BA (1996). Long-period volcano seismicity: Its source and use in eruption forecasting. Nature.

[8] Bean C, De Barros L, Lokmer I (2014). Long-period seismicity in the shallow volcanic edifice formed from slow-rupture earthquakes. Nat. Geosci..

[9] Harrington RM, Brodsky EE (2007). Volcanic hybrid earthquakes that are brittle-failure events. Geophys. Res. Lett..

[10] Matsubara W (2004). Distribution and characteristics in waveforms and spectrum of seismic events associated with the 2000 eruption of Mt. Usu. Earth Planet. Sci. Lett..

[11] Lokmer I, Saccorotti G, Di Lieto B, Bean CJ (2008). Temporal evolution of long-period seismicity at Etna Volcano, Italy, and its relationships with the 2004, 2005 eruption. Earth Planet. Sci. Lett..

[12] De Barros L (2011). Source Mechanism of Long Period events recorded by a high density seismic network during the 2008 eruption on Mt Etna. J. Geophys. Res..

[13] Nakano M, Kumagai H, Chouet BA (2003). Source mechanism of long-period events at Kusatsu-Shirane Volcano, Japan, inferred from waveform inversion of the effective excitation functions. J. Volcanol. Geotherm. Res..

[14] O’Brien GS (2011). Time reverse location of seismic long-period events recorded on Mt Etna. Geophys. J. Int..

[15] Bean CJ, Lokmer I, O’Brien GS (2008). Influence of near-surface volcanic structure on long-period seismic signals and on moment tensor inversions: Simulated examples from Mount Etna. J. Geophys. Res..

[16] De Barros L (2009). Source geometry from exceptionally high resolution longperiod event observations at Mt. Etna during the 2008 eruption. Geophys. J. Int..

[17] Elders WA, Friðleifsson GÓ, Pálsson B (2014). Iceland Deep Drilling Project: The first well, IDDP-1, drilled into magma. Geothermics.

[18] Koulakov I, Shapiro N, Beer M, Kougioumtzoglou I, Patelli E, Au IK (2021). Seismic tomography of volcanoes. Encyclopedia of Earthquake Engineering.

[19] Kim D, Brown LD, Árnason K, Gudmundsson Ó, Ágústsson K, Flóvenz ÓG (2020). Magma “bright spots” mapped beneath Krafla, Iceland, using RVSP imaging of reflected waves from microearthquakes. J. Volcanol. Geotherm. Res..

[20] Shalev-Shwartz S, Ben-David S (2014). Understanding Machine Learning: From Theory to Algorithms.

[21] Naeini EZ, Prindle K (2018). Machine learning and learning from machines. Lead. Edge.

[22] Röth G, Tarantola A (1994). Neural networks and inversion of seismic data. J. Geophys. Res..

[23] Langer H, Nunnari G, Occhipinti L (1996). Estimation of seismic waveform governing parameters with neural networks. J. Geophys. Res..

[24] Langer, H., Falsaperla, S. & Hammer, C. Advantages and pitfalls of pattern recognition, selected cases in geophysics. In *Volume 3, Computational Geophysics* 350 (Elsevier, 2020).

[25] Jia Y, Ma J (2017). What can machine learning do for seismic data processing? An interpolation application. Geophysics.

[26] Guitton, A., Wang, H. & Trainor-Guitton, W. Statistical imaging of faults in 3D seismic volumes using a machine learning approach. In *87th SEG Meeting. Houston, Texas, USA, Expanded Abstracts* 2045–2049 (2017).

[27] Turquais P, Asgedom EG, Söllner W (2017). Coherent noise suppression by learning and analysing the morphology of the data. Geophysics.

[28] Chen Y (2018). Automatic micro-seismic even picking via unsupervised machine learning. Geophys. J. Int..

[29] Lowney, B., Lokmer, I., O’Brien, G. S., Bean, C. J. & Igoe, M. Multi-domain diffraction identification using deep learning. In *81st EAGE Conference and Exhibition* (2019).

[30] Li, Z., Kovachki, N. B., Azizzadenesheli, K., Liu, B., Bhattacharya, K., Stuart, A. & Anandkumar, A. Fourier neural operator for parametric partial differential equations. arXiv 10.48550/arXiv.2010.08895 (2021).

[31] Zhang K, Zuo Y, Zhao H, Ma X, Gu J, Wang J, Yang Y, Yao C, Yao J (2022). Fourier neural operator for solving subsurface oil/water two-phase flow partial differential equation. SPE J..

[32] O’Brien GS, Bean CJ (2004). A 3D discrete numerical elastic lattice method for seismic wave propagation in heterogeneous media with topography. Geophys. Res. Lett..

[33] Yang Y, Gao AF, Castellanos JC, Ross ZE, Azizzadenesheli K, Clayton RW (2021). Seismic wave propagation and inversion with neural operators. Seism. Rec..

[34] Paszke, A., Gross, S., Massa, F., Lerer, A., Bradbury, J., Chanan, G. *et al.* PyTorch: An imperative style, high-performance deep learning library. In *Advances in Neural Information Processing Systems* 8024–8035 (Curran Associates, Inc., 2019). Available from: http://papers.neurips.cc/paper/9015-pytorch-an-imperative-style-high-performance-deep-learning-library.pdf

[35] Nooshiri N, Bean CJ, Dahm T, Grigoli F, Kristjánsdóttir S, Obermann A, Wiemer S (2022). A multibranch, multitarget neural network for rapid point-source inversion in a microseismic environment: Examples from the Hengill Geothermal Field, Iceland. Geophys. J. Int..

